# Transcriptomic analysis of *dead end* knockout testis reveals germ cell and gonadal somatic factors in Atlantic salmon

**DOI:** 10.1186/s12864-020-6513-4

**Published:** 2020-01-30

**Authors:** Lene Kleppe, Rolf Brudvik Edvardsen, Tomasz Furmanek, Eva Andersson, Kai Ove Skaftnesmo, Frida Thyri Segafredo, Anna Wargelius

**Affiliations:** 1 Institute of Marine Research, P.O. Box 1870, Nordnes, NO-5817 Bergen, Norway; 2 Cflow Fish Handling AS, Holsneset 25, N-6030, Langevåg, Norway

**Keywords:** Atlantic salmon, Gonadal somatic factors, *Gsdf*, *Inha*, Sertoli cell, Granulosa cell

## Abstract

**Background:**

Sustainability challenges are currently hampering an increase in salmon production. Using sterile salmon can solve problems with precocious puberty and genetic introgression from farmed escapees to wild populations. Recently sterile salmon was produced by knocking out the germ cell-specific *dead end* (*dnd*). Several approaches may be applied to inhibit Dnd function, including gene knockout, knockdown or immunization. Since it is challenging to develop a successful treatment against a gene product already existing in the body, alternative targets are being explored. Germ cells are surrounded by, and dependent on, gonadal somatic cells. Targeting genes essential for the survival of gonadal somatic cells may be good alternative targets for sterility treatments. Our aim was to identify and characterize novel germ cell and gonadal somatic factors in Atlantic salmon.

**Results:**

We have for the first time analysed RNA-sequencing data from germ cell-free (GCF)/*dnd* knockout and wild type (WT) salmon testis and searched for genes preferentially expressed in either germ cells or gonadal somatic cells. To exclude genes with extra-gonadal expression, our dataset was merged with available multi-tissue transcriptome data. We identified 389 gonad specific genes, of which 194 were preferentially expressed within germ cells, and 11 were confined to gonadal somatic cells. Interestingly, 5 of the 11 gonadal somatic transcripts represented genes encoding secreted TGF-β factors; *gsdf*, *inha*, *nodal* and two *bmp6-like* genes, all representative vaccine targets. Of these, *gsdf* and *inha* had the highest transcript levels. Expression of *gsdf* and *inha* was further confirmed to be gonad specific, and their spatial expression was restricted to granulosa and Sertoli cells of the ovary and testis, respectively. Finally, we show that *inha* expression increases with puberty in both ovary and testis tissue, while *gsdf* expression does not change or decreases during puberty in ovary and testis tissue, respectively.

**Conclusions:**

This study contributes with transcriptome data on salmon testis tissue with and without germ cells. We provide a list of novel and known germ cell- and gonad somatic specific transcripts, and show that the expression of two highly active gonadal somatic secreted TGF-β factors, *gsdf* and *inha*, are located within granulosa and Sertoli cells.

## Background

Atlantic salmon is a highly valuable commercial species, to which a substantial amount of research has been dedicated during the recent years. One of the bottlenecks currently limiting the possibilities to further increase the production is the environmental concern about farmed escapees breeding with wild populations of salmon, thereby causing genetic introgression [1, 2]. One solution would be the use of sterile salmon in aquaculture, and a germ cell-free sterile salmon (GCF salmon) has indeed been produced by either knockout or knockdown of the *dead end* (*dnd*) gene [3, 4]. Ongoing studies are investigating the possibility to block the function of Dnd in salmon at the RNA level (gene knockdown) in a similar way that has been done in zebrafish (*Danio rerio*) [5, 6]. Since it is challenging to develop a successful sterility treatment against a gene product already present in the body, alternative novel targets are also being investigated. Studies on salmon have identified and characterized genes preferentially expressed in germ cells [7–9], which could potentially serve as additional targets for the blocking of germ cell development. Another possible approach is to target proteins essential for germ cell survival or fertility. Such proteins could potentially be expressed in the germ cells, such as Bmp15 or Gdf9 [10–13]. However, the somatic gonad may also confer germ cell survival and fertility through specialized cells like granulosa and Theca cells (ovaries), and Sertoli and Leydig cells (testis), that are nurturing the germ cells and provide germ cell survival signals such as Dmrt1 [14]. Thus, blocking the function of proteins located in these nurturing cells or in the germ cells could potentially lead to sterility.

To be able to efficiently target proteins needed for germ cell survival or fertility, it is necessary, unless applying conditional knockout techniques, to target those genes that have an exclusive function limited to the gonad of the fish. As for now, some information is available on genes exclusively expressed in germ cells in Atlantic salmon [7–9], and only one study has so far revealed a function of such proteins in salmon [3]. Limited information is available for genes expressed exclusively in the somatic gonad of salmon. The most known is the sex determining gene *sdy*, which is expressed in the male somatic gonad [15, 16]. It is also known that the gonadotropin receptors *follicle-stimulating hormone receptor* (*fshr*) and *luteinizing hormone receptor* (*lhcgr*) are predominantly expressed in the gonads of salmon, and in females the expression is restricted to granulosa and Theca cells [17]. However, a weak expression of *fshr* and *lhcgr* have also been found in extra-gonadal tissues like gills, brain, liver and heart [18]. The *nuclear progesterone receptor* (*pgr*) is mainly expressed in testis tissue, but also ovary and weakly in pituitary and spleen. Within the testis, *pgr* transcripts are restricted to Sertoli cells [19]. Aromatase (Cyp19a1a) has been located to germ and somatic cells in testis tissue from one year old males [20]. Furthermore, *cyp19a1a* transcripts have been detected in GCF and WT ovaries, while not detected in 7 other adult tissues. A similar expression profile has been shown for *anti-müllarian hormone* (*amh*) [3]. The same study reported expression of *forkhead box L2* (*foxl2*) and *sex-determining region Y-box 9a* (*sox9a*) in both GCF and WT ovary and testis tissue, however von Schalburg et al. [21] have shown that both *foxl2* and *sox9a* are also expressed in a number of extragonadal adult tissues. Additional genes that are associated with gonadal functions, such as for example *insulin-like growth factor 3* (*igf3*), have also been detected in both GCF and WT gonads of salmon [22]. Nevertheless, information is currently lacking on potential extra-gonadal expression and additional functions of such genes.

The GCF Atlantic salmon model represents a unique opportunity to identify genes that are exclusively expressed in gonadal somatic cells in this species. The GCF salmon male model contains a high number of Sertoli cells [3], which makes it likely to identify Sertoli cell specific genes. To identify unique somatic and germ cell factors, we performed transcriptome sequencing of gonad tissue (testis) from male wild type (WT) and GCF salmon. Our dataset was then compared with available multi-tissue transcriptome data (GenBank GBRB00000000.1 [23];) to eliminate genes with extra-gonadal expression. This study therefore presents more germ cell specific genes, which adds to the current group of known germ cell specific genes in salmon and opens for further research on potential sterility targets. Furthermore, we have provided a list of gonadal somatic genes and characterized the expression patterns of the two most highly expressed gonadal somatic genes in salmon, *gsdf* and *inha*.

## Results

### Gonad specific genes preferentially expressed in gonadal somatic cells

In silico filtering criteria applied in this study were based on a previous similar study [9] and are discussed below. To first identify genes expressed specifically in salmon gonads we searched available multi-tissue transcriptome sequencing data (GenBank GBRB00000000.1 [23];) for genes with 50 or less reads in extra-gonadal tissues. Further, genes with less than 100 reads in testis or ovary were excluded from the analysis. According to these criteria, 389 unique gene ID’s were identified (Additional file 1). Several genes such as *deleted in azoospermia like* (*dazl*) (GenBank 106,588,867), *dead end* (*dnd*) (GenBank 101,448,053) *and piwi like RNA-mediated gene silencing 1* (*piwil1*) (GenBank 106,585,526), well-known to be expressed preferentially in gonads, were identified in this list (Additional file 1).

**Fig. 1:**
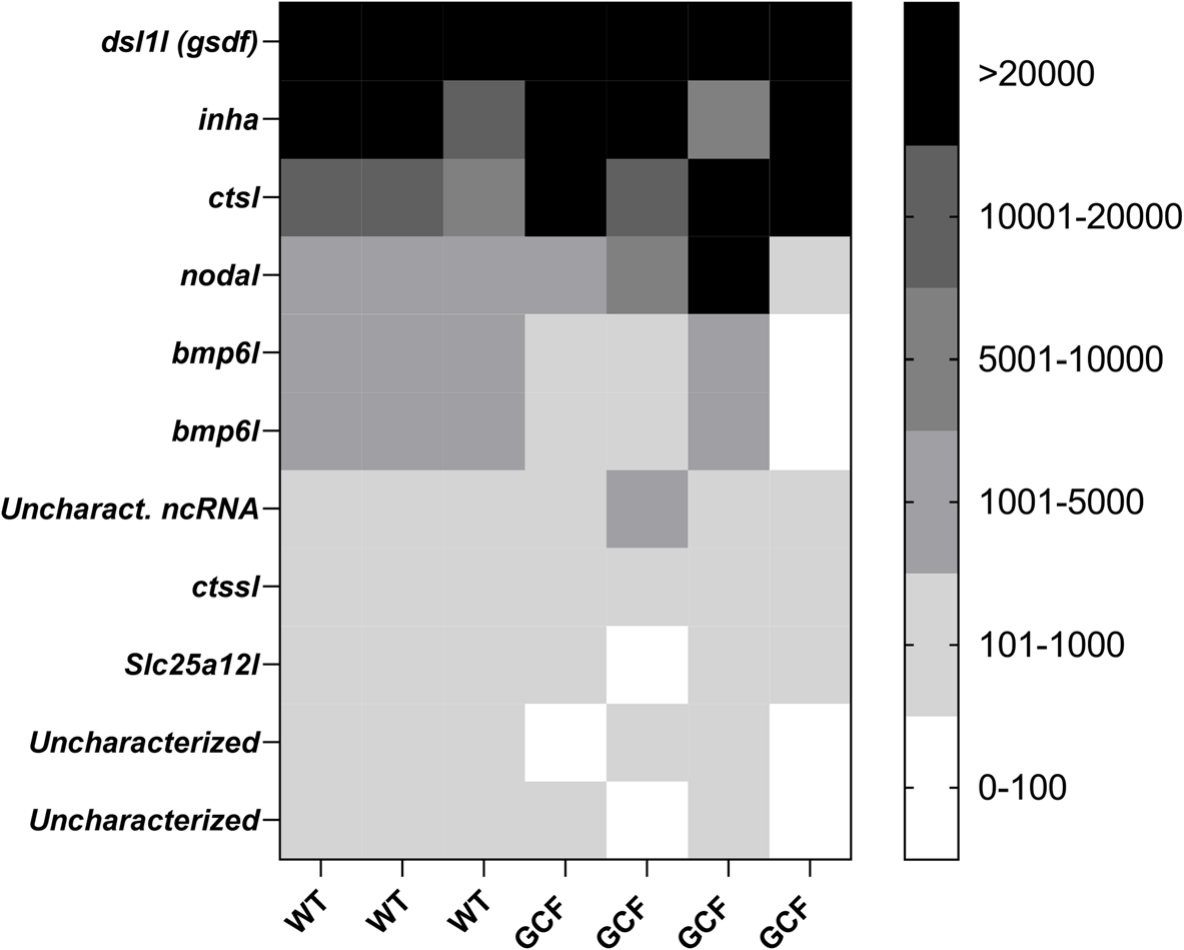
Expression profiles of 11 gonadal somatic genes. Expression profiles, shown as number of reads, of 11 genes with expression confined to gonadal somatic cells in adult immature Atlantic salmon testis tissue. WT; wild type testis (*n* = 3), GCF; germ cell-free testis (*n* = 4)

Next, the 389 identified sequences were merged with the sequences obtained from GCF and WT testis to reveal which genes that were expressed in testicular somatic cells. Eleven genes had 100 or more reads in both GCF and WT testis (Fig. 1; Table 1). Of these, two genes and one noncoding RNA have not been characterized. Of the known genes, *solute carrier family 25 member 12-like* (*slc25a12l*) and *cathepsin s-like* (*ctssl*) had the lowest numbers of reads (between ~ 200–600 reads). Another cathepsin – *cathepsin L* (*ctsl*) – had a noteworthy higher expression with ~ 12,000–22,000 reads. The remaining genes – two *bone morphogenetic protein 6-like* (*bmp6l*) genes, *nodal*, *protein inhibin alpha chain* (*inha*) and *gonadal somatic-derived factor* (*gsdf*) – which all belong to the TGF-β superfamily, had highly variable expression levels ranging from ~ 1000–2000 reads (*bmp6l*) and ~ 2000–8000 reads (*nodal*) to more than 22,000 reads (*inha* and *gsdf*). Another noteworthy observation is the clear difference in number of transcripts of *nodal* (~ 3-fold increase) and *gsdf* (~ 3,5-fold decrease) from WT to GCF testis. The two genes with the highest expression in testicular somatic cells (and therefore potentially have a high activity in these cell types), *inha* and *gsdf*, were selected for further characterization in salmon males and females.

**Table 1:** Gonad specific genes with expression in testicular somatic cells of Atlantic salmon

Gene ID	Average reads in WT testis	Average reads in GCF testis	Annotation
106,568,506	248,942.4	71,175.8	*dsl1l (gsdf)*
106,575,603	21,784.4	24,951.3	*inha*
106,561,951	12,028.3	21,845.6	*ctsl*
106,580,334	2616.9	7858.6	*nodal*
106,585,325	2658.0	1028.6	*bmp6l*
106,585,642	2649.9	1021.4	*bmp6l*
106,610,757	357.3	843.9	*Uncharacterized ncRNA*
106,561,959	247.0	463.1	*ctssl*
106,600,069	634.1	202.9	*Slc25a12l*
106,609,789	698.5	171.8	*Uncharacterized*
106,578,524	145.7	125.1	*Uncharacterized*

Gonad specific (≤50 reads in extragonadal tissues) genes with expression (≥100 reads) in testicular somatic cells of Atlantic salmon. Gene ID, average number of reads in wild type (WT) and germ cell-free (GCF) testis, and annotation are shown.

### Gonad specific genes preferentially expressed within germ cells

**Fig. 2:**
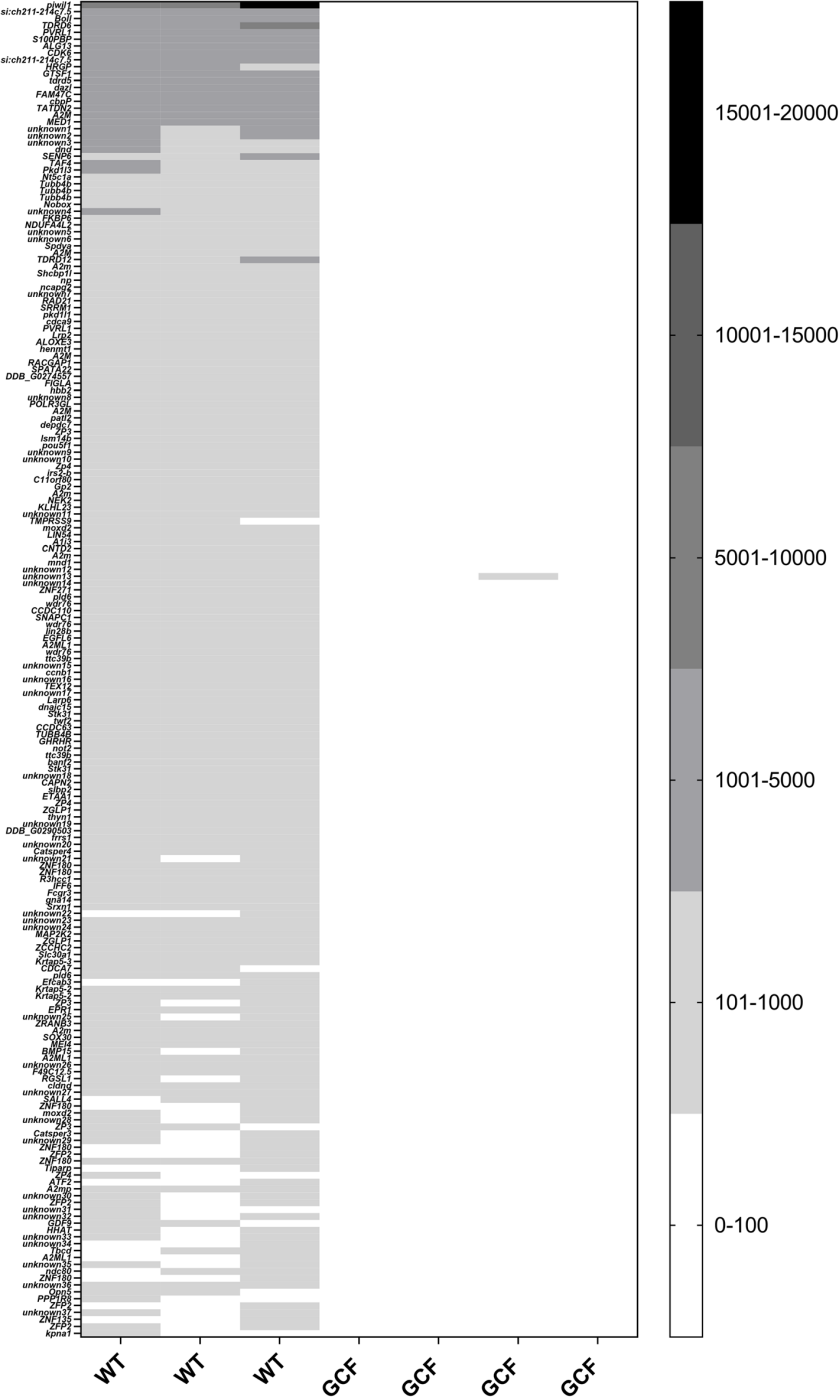
Expression profiles of 194 germ cell specific genes. Expression profiles, shown as number of reads, of 194 genes with expression confined to germ cells in adult immature Atlantic salmon testis tissue. WT; wild type testis (*n* = 3), GCF; germ cell-free testis (*n* = 4)

To identify genes that are preferentially expressed in salmon germ cells, the gonad specific 389 genes were filtered again based on number of reads in WT and GCF testis; genes with more than 50 reads in the GCF group, and transcripts with less than 100 reads in the WT group, were excluded. Based on these criteria, 194 genes were expressed in WT testis and not GCF testis (Fig. 2). Several well-known germ cell-specific genes like *piwil1* (GenBank 106,585,526), *dazl* (GenBank 106,588,867) and *tudor domain containing 6* (*tdrd6*) (GenBank 106,608,113) could be found in this list of 194 genes [Fig. 2; (Additional file 2)]. Among these, 69 genes were annotated with KEGG pathway ID’s (when removing human diseases). The pathway with the highest number (14) of genes was lysine degradation, followed by complement and coagulation cascades (13 genes). The pathways linked to more than one gene is shown in Table 2.

**Table 2:** KEGG pathways linked to gonad specific genes preferentially expressed in germ cells

Pathway ID	Pathway	Number of genes
310	Lysine degradation	14
4610	Complement and coagulation cascades	13
4540	Gap junction	5
4145	Phagosome	4
4110	Cell cycle	4
230	Purine metabolism	3
240	Pyrimidine metabolism	3
4068	FoxO signaling pathway	3
4022	cGMP-PKG signaling pathway	3
4151	PI3K-Akt signaling pathway	3
4514	Cell adhesion molecules (CAMs)	3
4914	Progesterone-mediated oocyte maturation	3
3020	RNA polymerase	2
4141	Protein processing in endoplasmic reticulum	2
4010	MAPK signaling pathway	2
4152	AMPK signaling pathway	2
4080	Neuroactive ligand-receptor interaction	2
4210	Apoptosis	2
4115	p53 signaling pathway	2
4520	Adherens junction	2
4550	Signaling pathways regulating pluripotency of stem cells	2
4621	NOD-like receptor signaling pathway	2
4910	Insulin signaling pathway	2
4915	Estrogen signaling pathway	2
4918	Thyroid hormone synthesis	2
4919	Thyroid hormone signaling pathway	2
4211	Longevity regulating pathway	2
1522	Endocrine resistance	2

Pathway ID, pathway and number of genes within each pathway are shown. Pathways associated with only one gene are not shown. The full list of KEGG pathways can be viewed in [Additional File 3]

### Phylogenetic analysis of the Inha protein sequence and chromosomal synteny of the *inha* gene

To confirm the identity of the two selected genes *dsl1 (gsdf, see below)* and *inha* (GenBank 106,568,506 and 106,575,603) in this study, a phylogenetic analysis and a search for chromosomal synteny was performed. However, in the case of the gene annotated to *dsl1l* (GenBank 106,568,506), a homology search had previously been performed using EST databases (GenBank CK897686.1 for salmon is identical to GenBank 106,568,506), suggesting that this gene is *gonadal somatic-derived factor* (*gsdf*) [24]. The same gene has also been studied in salmon by Lubieniecki et al. [25].

**Fig. 3:**
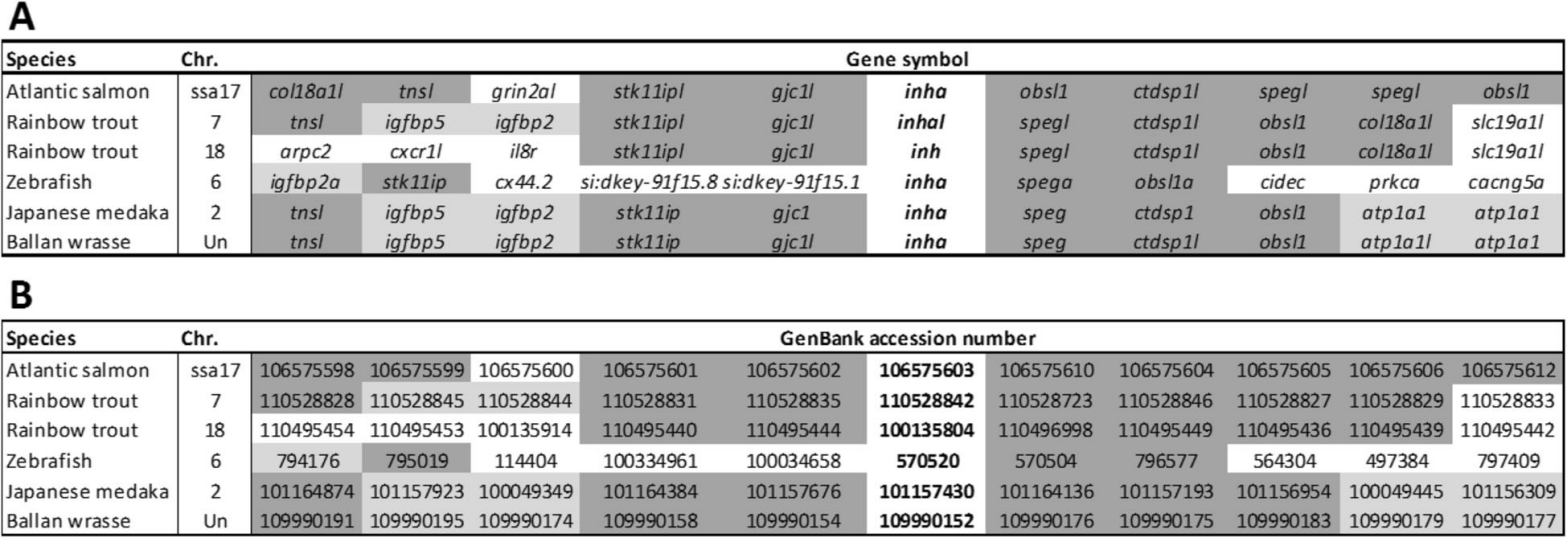
Synteny of chromosomal regions associated with *inha*. Overview of the 5 most adjacent up-and downstream genes to *inha* (**a**; gene symbols, **b**; gene IDs) in a selection of fish species, including Atlantic salmon, is shown. Genes marked with dark grey color are conserved in salmon and the other species. Genes marked with light grey color are conserved in other species than salmon. Chr; Chromosome

In the case of *inha*, a phylogenetic analysis (Additional file 4) was performed by comparing Inha protein sequences from tetrapods, teleosts and Spotted gar, a ray-finned fish that diverged from the teleosts [26]. To further confirm the identity of *inha*, we investigated the synteny of chromosomal regions associated with this gene in a selection of fish species. We searched for genes located up- and downstream of *inha*, and conserved genes were identified in all the selected species (Fig. 3). In Atlantic salmon, 3 additional *inha*-like genes were found, however they are annotated as, and appear to be, pseudo genes (GenBank Accession no 106574595; 106,574,698; 106,596,067).

### Expression of *gsdf* and *inha* in Atlantic salmon tissues

**Fig. 4:**
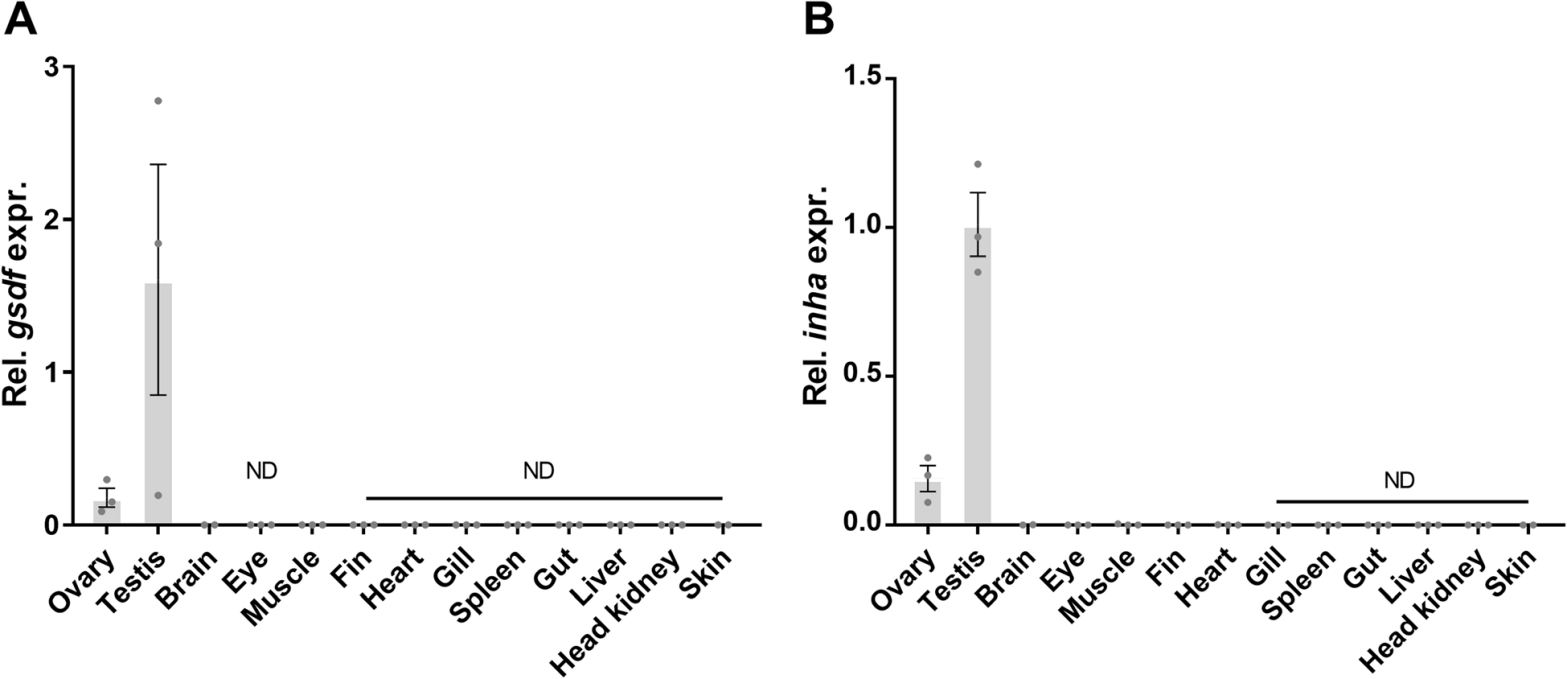
Relative expression of *gsdf* and *inha* – tissue distribution in adult immature salmon. Expression of *gsdf* **a** and *inha* **b** relative to *EF1α*, in different tissues from immature adult Atlantic salmon, measured by qPCR. All values were calibrated to the average ΔCt of the group with the highest expression (testis). The data are shown as mean with SEM (*N* = 2–3 individuals). ND, not detected

A qPCR tissue screen was performed to confirm that *gsdf* and *inha* are specifically expressed in gonads of Atlantic salmon. As shown in Fig. 4, *gsdf* (A) and *inha* transcripts (B) are abundant in ovary and testis, and very low or undetected in all other tissues examined. Although the statistical test applied detected significant differences between groups, the post-test was not able to distinguish which groups that were significantly different; 3 biological replicates were used per tissue.

**Fig. 5:**
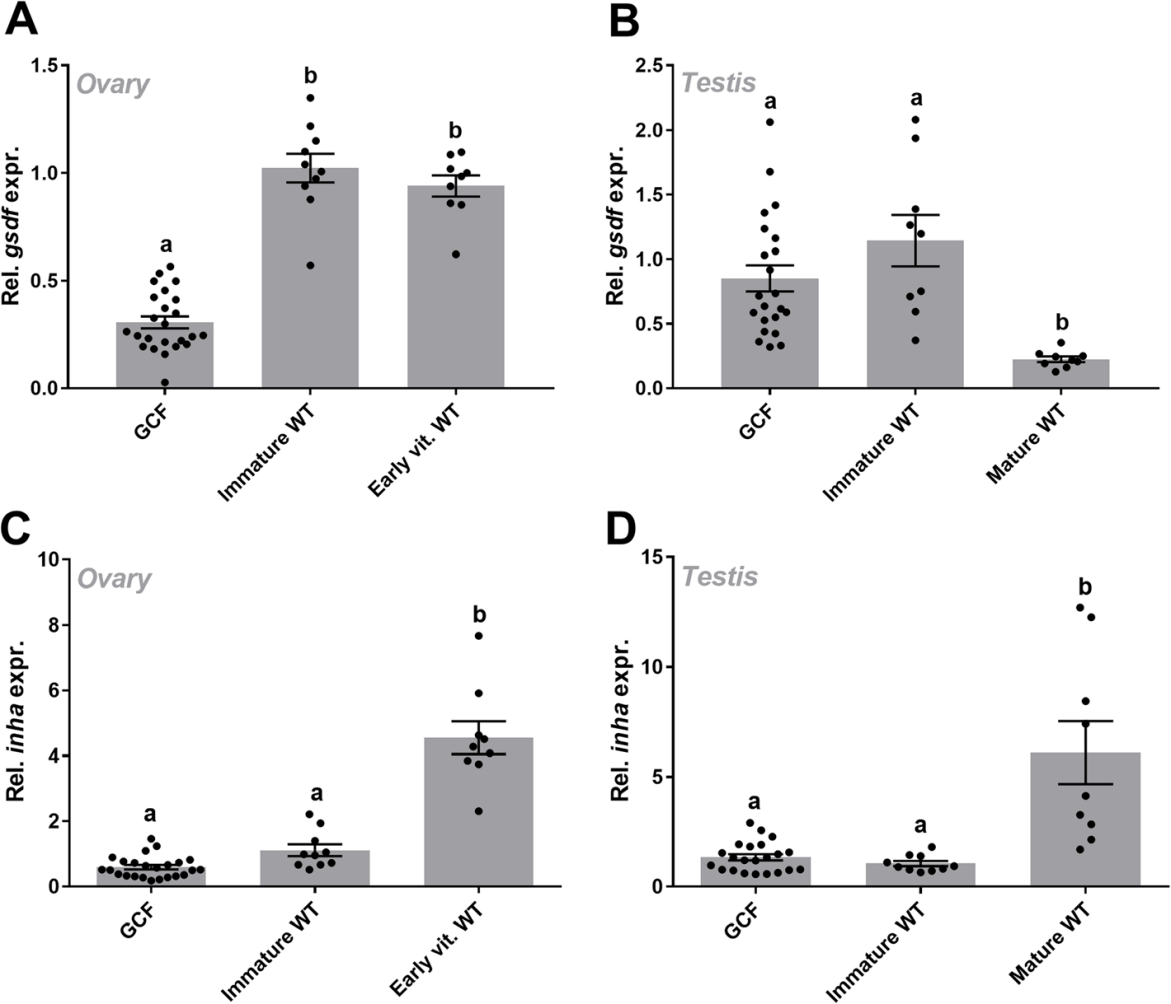
Relative expression of *gsdf* and *inha* in germ cell-free and wild type gonads. Expression of *gsdf* **a**, **b** and *inha* **c**, **d** relative to *EF1α*, in GCF and two stages of WT ovary **a**, **c** and testis **b**, **d** of Atlantic salmon, measured by qPCR. All values were calibrated to the average ΔCt of the immature WT group. The data are shown as mean with SEM (*N* = 9–24 individuals). Different letters represent significant differences between groups (*p* < 0.05). GCF, germ cell-free; WT, wild type

Transcript levels of *gsdf* and *inha* were further measured in GCF and WT ovary and testis. As shown in Fig. 5, *gsdf* and *inha* transcripts were detected in both GCF and WT ovary (A, C) and testis (B, D). Further, we observed that *gsdf* expression was higher in WT than GCF ovary tissue, and that the expression remained unchanged from the immature to the early vitellogenic group (Fig. 5a). In testis tissue, *gsdf* was downregulated from immature to mature samples (Fig. 5b). Expression of *inha* was upregulated in early vitellogenic ovary and mature testis (Fig. 5c, d).

**Fig. 6:**
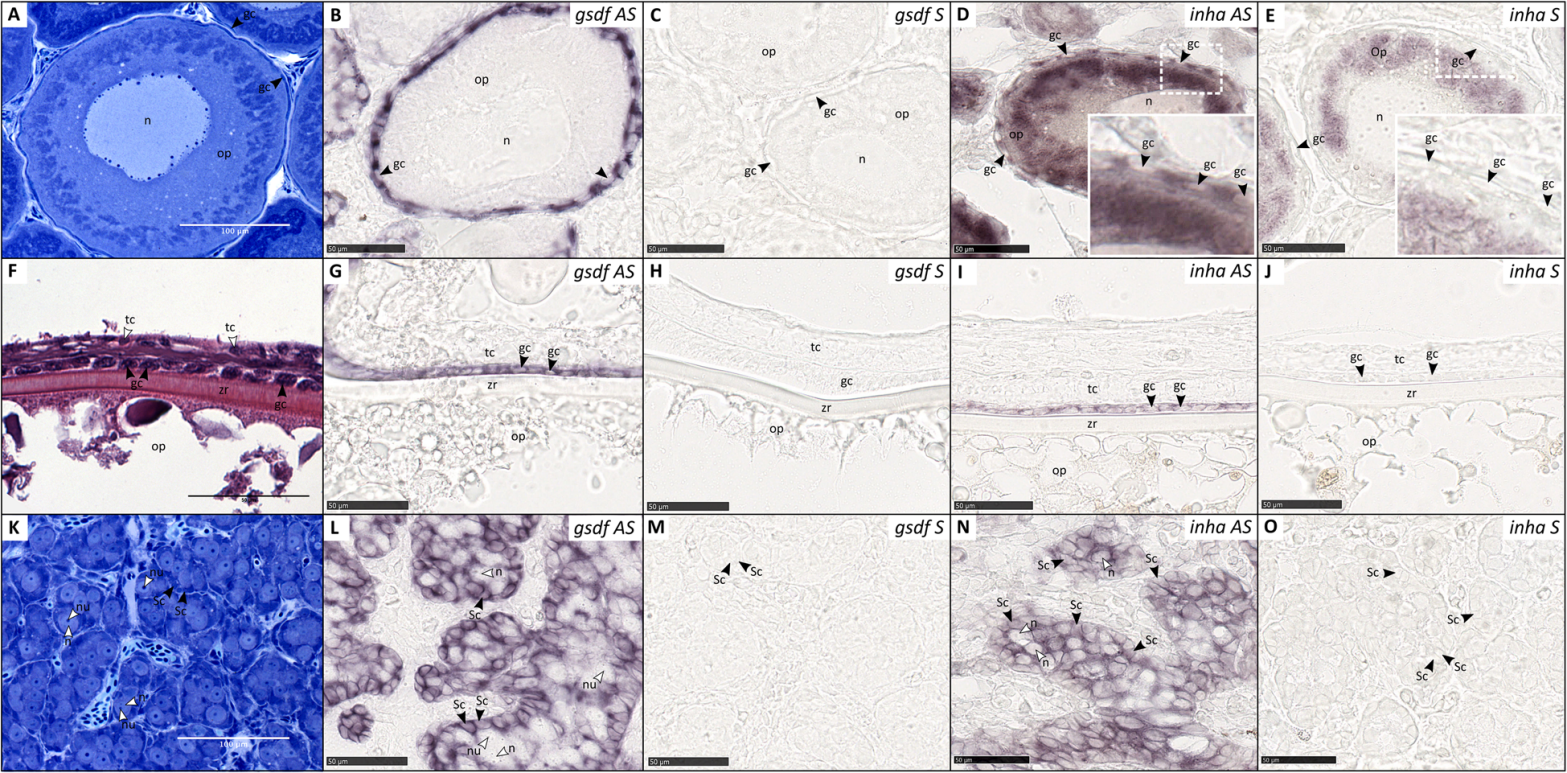
Gene expression of *gsdf and inha* – localized to granulosa and Sertoli cells. Expression of *gsdf* and *inha* in Atlantic salmon ovary (*gsdf*: **b**, **c**, **g**, **h**; *inha*: **d**, **e**, **i**, **j**) and testis (*gsdf*: **l**, **m**; *inha*: **n**, **o**), visualized by in situ hybridization. Germ cell and gonadal somatic cell structures are shown by Toluidine Blue stained plastic sections of immature ovary **a** and testis tissue **k**, and a Hematoxylin Eosin Saffron stained paraffin section of a vitellogenic oocyte **f**. Expression of *gsdf* and *inha* is represented by the staining in images where the antisense (AS) probe was applied. **b**-**e** Immature ovary. **g**-**j** Vitellogenic ovary. **l**-**o** Immature testis. Gc, granulosa cells; op, ooplasm; tc, theca cells; zr, zona radiata; Sc, Sertoli cell; n, nucleus of spermatogonia; nu, nucleoli of spermatogonia; S, sense probe

To investigate the gonadal location of *gsdf* and *inha* transcripts in salmon, in situ hybridization (ISH) was performed on both ovary and testis tissue. In females, *gsdf* and *inha* transcripts were detected in granulosa cells (Fig. 6b, d), which was also confirmed in females with larger, vitellogenic oocytes (Fig. 6g, i). In the case of *inha*, we also observed some staining within the ooplasm of immature oocytes (Fig. 6d), however, a similar staining was present in immature oocytes where the sense probe was applied (Fig. 6e) and is therefore considered as unspecific staining. In males, *gsdf* and *inha* were expressed in Sertoli cells (Fig. 6l, n).

## Discussion

In this study we have made available transcriptome data on genes expressed in the germ cell-free (GCF) Atlantic salmon testis tissue, which can also be compared to genes that are expressed in the same type of tissue with germ cells present. We have from these data identified genes that are preferentially expressed in both gonadal somatic cells (Fig. 1; Table 1) and germ cells of salmon [Fig. 2; (Additional file 2)], which adds novel factors to the list of genes that have been reported previously [7–9]. This dataset therefore provides valuable information for further studies on potential sterility targets in Atlantic salmon, but also serves as an interesting resource for the field of reproductive biology in a wider context, such as cell-cell communication within the testis.

### Validation of tissue specificity of the identified transcripts

In this study we characterized in more detail two genes with a high expression (read counts) in gonadal somatic cells, *gsdf* and *inha*. QPCR analysis showed that both these genes are expressed in ovary and testis tissue lacking germ cells, confirming their expression in gonadal somatic cells. ISH analysis showed that *gsdf* and *inha* transcripts are localized to granulosa cells in females and in Sertoli cells in males, confirming their specific gonadal somatic expression. A similar expression pattern has recently been shown in salmon testis tissue for another gonadal somatic gene, *anti-müllarian hormone* (*amh*) [27], which supports our findings. In the case of germ cell-specific genes identified in this study, several of these including *piwil1*, *dazl*, *la-related protein 6-like* (*larp6l*), *bone morphogenetic protein 15-like* (*bmp15l*) and *folliculogenesis specific bHLH transcription factor* (*figla*) have been validated by us using qPCR and ISH in previous publications [8, 9].

### In silico filtering thresholds

To be able to say that a gene is expressed or not in a given tissue sample, a threshold for number of reads needs to be determined. In this study we applied the same thresholds as a previous similar study [9]; firstly, genes with 50 or less reads are not considered expressed, while fewer reads are considered as background/noise. This choice is supported by the fact that for example *vasa*, known to be expressed exclusively in germ cells, still had 70 and 57 reads in eye and gill tissues. Secondly, we chose 100 reads as a minimum for genes considered to be expressed; this creates a clear distinction from genes considered not expressed (50 or less reads). When applying these criteria for in silico filtering, several well-known 1) gonad-specific genes were identified in the list of gonad-specific genes [Additional File 1], 2) well-known germ cell specific and gonadal somatic specific genes were identified in the lists of genes expressed in germ cells [Additional File 2] and in gonadal somatic cells (Table 1), which validates the chosen thresholds. Nevertheless, cutoffs most likely also cause some relevant genes to be excluded from the analysis.

### Genes with expression preferentially in gonadal somatic cells

To our knowledge, this is the first study to screen for genes with expression exclusively in gonadal somatic cells in Atlantic salmon, using a knockout model. Only 11 genes (*gsdf*, *inha*, *ctsl*, *nodal*, two *bmp6l*, *ctssl*, *slc25a12l*, two unknown genes and one unknown ncRNA) had 100 or more reads in GCF and WT testis (Table 1) while at the same time being exclusive to gonadal tissue. The most striking finding was that around half of these genes, (*gsdf*, *inha*, *nodal* and the two *bmp6l*) are associated with the TGF-β pathway. They also represented the two genes with the highest expression in testicular somatic cells, *gsdf* and *inha*, two genes that we have studied and discussed in more detail in the next sections. TGF-β proteins are all secreted ligands known to be essential for many processes in gonad development including diverse functions in the testis associated with germ cells, Sertoli cells, growth and fertility [28]. However in our case, the finding of TGF-β transcripts restricted to gonadal somatic cells is truly promising for future targeting since TGF-β proteins are secreted proteins that may likely have functions confined to the gonad, which is a prerequisite for 1) functional studies of the gonad without using conditional mutants, and 2) finding potential target proteins for a sterility vaccine in salmon. Interestingly, *bmp6* plays a role in human Sertoli cell proliferation and apoptosis [29]. In fish, Bmp6 has been linked to fin regeneration, iron metabolism, tooth patterning and viability and growth [30–33]. Furthermore, *bmp6* has been linked to ovarian function in tounge sole and zebrafish [34, 35]. The diverse roles of this protein may be explained by its widespread expression in many tissues, however our finding of a unique *bmp6*-like transcript confined largely to the somatic gonad may be a result of sub-functionalization and a specialized function of this protein in the gonad of salmon. Nodal, another TGF-β protein, is involved in various processes within mammalian testes. More specifically, Nodal signaling plays a role in regulation of pluripotency factor expression, proliferation and survival of germ cells, as well as establishment of the somatic niche through seminiferous cord formation, steroidogenesis and Sertoli cell function (reviewed by [36]). In human testis, Nodal has been shown to regulate Sertoli cell proliferation [37]. In zebrafish Nodal is involved in dorso-ventral patterning of the embryo [38], while to our knowledge no specific studies have investigated the function of this protein in the fish gonad. A high *nodal* expression restricted to gonads in salmon as observed in this study suggests a role of this gene in reproduction in fish. Interestingly, we observed a 3-fold higher *nodal* expression in salmon testis lacking germ cells, compared to WT testis. This may indicate that the presence of germ cells has an inhibitory effect on *nodal* expression in Sertoli cells of immature testis tissue, and that Nodal is part of the germ-somatic cell communication in Atlantic salmon. Two Cathepsins, Ctsl and Ctssl, had gonad specific transcripts and were also expressed within testicular somatic cells in salmon. These are Cysteine Cathepsins, proteases involved in a number of physiological processes like protein breakdown and immune responses [39, 40]. Interestingly, the stage-specific expression of *ctsl* in rat Sertoli cells is influenced by the presence or absence of germ cells, suggesting a role of *ctsl* in germ-somatic cell interaction in testis tissue [41]. A higher number of *ctsl* as well as *ctssl* reads in GCF compared to WT testis tissue as observed in this study, may suggest possible involvement for these Cathepsins in germ-somatic cell signaling in salmon.

### Gsdf

The identity of Atlantic salmon *gsdf* has previously been shown by phylogenetic analysis [24]. In this study we found that *gsdf* is highly expressed in females and males, with specific transcript localization in granulosa and Sertoli cells, respectively. Likewise, expression of *gsdf* in the same cell types has been observed in several fish species including rainbow trout (*Oncorhynchus mykiss*), medaka (*Oryzias latipes*), zebrafish, Nile tilapia (*Oreochromis niloticus*), Olive flounder (*Paralichthys olivaceus*), Spotted scat (*Scatophagus argus*) and Japanese flounder (*Paralichthys olivaceus*) [24, 42–48]. Transcripts of *gsdf* have also been detected within oocytes in Olive flounder, and within spermatogonia and spermatids in Chinese tongue sole (*Cynoglossus semilaevis*) [46, 49]. Although the function(s) of *gsdf* in male fish seems strongly associated with germ cells, it may differ between species since this gene has been linked to several processes such as proliferation of primordial germ cells and spermatogonia in rainbow trout, sex determination in *Oryzias luzonensis*, sex differentiation in Chinese tounge sole, and testis differentiation in medaka and Nile tilapia [24, 49–53]. Although we do not know the function of Gsdf in salmon testis, it is likely that this protein has one or more roles as described in other fish species. One possible role may be associated with testis maturation, since we observed that *gsdf* expression was higher in immature compared to mature testis, similar to what has been observed in medaka, Japanese flounder, wrasse (*Halichoeres trimaculatus*) and rice field eel (*Monopterus albus*) [43, 48, 54, 55]. The fact that we detected a 3.5-fold lower number of *gsdf* reads in testis tissue devoid of germ cells compared to intact testis tissue may suggest a role for *gsdf* in the communication between germ cells and testicular somatic cells in Atlantic salmon.

In salmon females we observed *gsdf* expression specifically localized to granulosa cells in both previtellogenic and vitellogenic follicles. The localization of *gsdf* expression in ovarian somatic cells was further confirmed by the presence of *gsdf* transcripts in GCF ovaries, although at a lower level compared to normal ovaries. Limited information exists on the role of Gsdf in females. It is known that targeted disruption of *gsdf* in medaka causes abnormal folliculogenesis associated with sterility [56]. In zebrafish, Gsdf may have a role in regulating ovarian follicle maturation and expression of genes involved in steroid biosynthesis, obesity, diabetes, and female fertility [57]. Lack of Gsdf in medaka has been linked to a dysregulation of oocyte development [58]. In coho salmon (*Oncorhynchus kisutch*) ovarian tissue, *gsdf* transcript levels are significantly increased during early secondary growth. The authors speculated that Gsdf may be involved in granulosa cell proliferation [59]. In contrast, we did not observe any increase in *gsdf* expression in early vitellogenic salmon ovaries. This may be explained by a difference in the maturation stage, since the rainbow trout ovaries in the previous study were more advanced than the salmon ovaries applied in the current study. Based on our and others’ findings, we speculate that salmon *gsdf*, due to its specific localization in granulosa cells, may be involved in folliculogenesis.

### Inha

In this study, Atlantic salmon *inha* was confirmed by chromosomal synteny to other fish species and by its close phylogenetic relationship within teleosts. Furthermore, we confirmed that *inha* expression is localized in granulosa and Sertoli cells of salmon ovary and testis, respectively. In agreement with our observations, studies in rainbow trout and zebrafish females have shown that *inha* expression is restricted to the somatic follicle cells surrounding the oocytes [60, 61]. Similarly, male rainbow trout *inha* expression is localized to Sertoli cells, although *inha* transcripts were also detected in Leydig cells [60]. In the current study it was difficult to distinguish Leydig cells from the clearly stained and numerous Sertoli cells; thus, a conclusion could not be made on whether the Leydig cells contained *inha* transcripts. Based on the similarities of our results to the above-mentioned studies we suggest a similar functional role for Inha in salmonids.

Further characterization of *inha* in salmon revealed that the transcript level increased during puberty in both females and males, suggesting a role in sexual maturation. In the case of females, this is supported by studies where *inha* expression increased during folliculogenesis and peaked during final oocyte maturation in zebrafish [61] and coho salmon [62]. Limited information exists on the function of Inha in teleost testis, however some insight has been generated from studies in rainbow trout. It was shown that testicular *inha* expression is upregulated by Fsh and Lh [63], and that the response in *inha* transcript levels to Fsh is mediated through the production of sex steroids [64]. This interaction of *inha* transcript levels with Fsh and sex steroids in trout, together with the localization of *inha* mRNA in Sertoli cells and increase of testicular *inha* expression during spermatogenesis (this study), suggest an essential role of Inha in spermatogenesis in salmonids.

### Genes with expression preferentially in germ cells

In our dataset we also had the opportunity to identify transcripts confined to germ cells, based on their expression in WT and absence in GCF testis. We cannot rule out however, that some of these genes may be expressed in gonadal somatic cells, but are downregulated as a response to the absence of germ cells. Nevertheless, numerous candidates were identified [Fig. 2; (Additional file 2)], including several transcripts encoding well-known germ cell specific proteins such as Piwi, Dazl and Tdrd6. KEGG pathways annotated to the genes revealed that several of them are involved in lysine degradation and complement and coagulation cascades (Table 2). Lysine degradation is a pathway essential for metabolic function in the cell and can in this way have a special function in germ cells. Another speculation of an enrichment of these pathways in the gonad containing germ cells may be a potentially higher content of blood vessels in gonads with germ cells, since these pathways are associated with blood activity.

## Conclusions

This study provides transcriptome data from salmon testis tissue with and without germ cells. By comparing these transcriptomes, we identified 389 gonad specific genes, of which 194 were preferentially expressed within germ cells, and 11 were confined to the somatic part of the gonad. Five of these 11 genes (*gsdf*, *inha*, *nodal* and two *bmp6-like* genes) encode TGF-β proteins. Among the gonadal somatic genes, *gsdf* and *inha* had the highest numbers of transcripts. Expression of *gsdf* and *inha* was restricted to ovarian granulosa and testicular Sertoli cells. While *inha* transcript levels increase during puberty in both females and males, expression of *gsdf* decreases from immature to mature males. Information on genes with functions in gonadal somatic cells will be useful in future studies with the aim to elucidate how germ cells are supported for development and survival. Ultimately, knowledge on how germ cells develop and stay alive may lead to the possibility to control gametogenesis in this commercially important species.

## Methods

### Animals, rearing and sampling of tissue

The use of the experimental animals in this study was performed in strict accordance with the Norwegian Animal Welfare Act of 19th of June 2009, in force from 1st of January 2010. All year classes of fish reared were approved by the Norwegian Animal Research Authority (http://www.fdu.no/fdu/NARA, permit number 5741. A completed ARRIVE guidelines checklist can be viewed in [Additional File 5]. All the fish were reared and sampled at Matre Aquaculture Research Station, Matredal, Norway. Four groups of fish were applied, of which groups 1–3 have been published in previous studies; consequently, only 3 experimental animals were sacrificed solely for the current study (group 4).

*Group 1*: 7 Atlantic salmon males, 1 year old, reared and sampled as described previously [3]. The following tissues were included for RNA sequencing to screen for genes expressed in gonadal somatic cells: GCF/*dnd*-knockout (*n* = 4) and WT testis (*n* = 3).

*Group 2*: 6 Atlantic salmon (3 females, 3 males), 1–2 years old, reared and sampled as described previously [9]. The following tissues were included for a qPCR analysis to screen for *inha* and *gsdf* expression in various parts of the body: ovary, testis, brain, eye, muscle, fin, heart, gill, spleen, gut, liver, head kidney, skin (Fig. 4).

*Group 3*: 83 Atlantic salmon (24 GCF/*dnd*-knockout, 10 immature and 9 early vitellogenic females; 22 GCF/*dnd*-knockout, 9 immature and 9 mature males), 2 years old, reared and sampled as described previously [22]. The following tissues were included for qPCR analysis to reveal if *inha* and *gsdf* expression in the gonads change through puberty: GCF, immature and early vitellogenic ovary, and GCF, immature and mature testis (Fig. 5). The selected gonadal stages have been described previously based on gonado-somatic index, plasma levels of sex steroids and histology [22].

*Group 4*: 3 Atlantic salmon (1 immature and 1 vitellogenic female; 1 immature male), reared in indoor tanks under standard rearing conditions and a maturation inducing regime [65]. The fish were fed ad libitum with a standard commercial diet. Prior to sampling, all fish were anesthetized with 2 ml/L finquel vet, and sacrificed by cutting into the medulla oblongata, the connection between the spinal cord and the skull. Gonad tissue was collected and fixed in 4% paraformaldehyde in PBS over-night at 4 °C. Subsequently, the samples were washed 2 × 2 hours in PBS, immersed in 25% sucrose in PBS, and stored over-night at 4 °C. Finally, the samples were embedded in Tissue Tek and stored in − 80 °C until ISH. The following tissues were included for ISH to reveal the cellular localization of *gsdf* and *inha* transcripts in the gonads: immature testis and ovary, and vitellogenic ovary (Fig. 6).

### In silico analysis and RNA sequencing

Firstly, to identify genes expressed specifically in salmon gonads, we screened available salmon multi-tissue (ovary, testis, brain, eye, gill, gut, head kidney, heart, liver, muscle, nose, ovary, pyloric caecum, skin, and spleen) transcriptome sequencing data (GenBank GBRB00000000.1 [23];). Genes with expression in extra-gonadal tissues were removed from the analysis.

Secondly, to identify which of the gonad specific genes that were expressed in the somatic part of the gonads, and which of them that were expressed within germ cells, we sequenced total RNA from 3 WT and 4 GCF testis (raw sequence reads accession no. PRJNA550414; https://www.ncbi.nlm.nih.gov/bioproject/PRJNA550414) using the HiSeq.2000 sequencing platform (Illumina). RNA-seq paired end sequences were mapped with Bowtie2 against the gene model transcripts of Atlantic salmon genome (ICSASG_v2) with standard Bowtie2 parameters [66]. Raw count table for each gene was extracted using SAMtools idxstats [67]. The read counts were normalized to the total reads in the sample with the smallest number of reads. A summary of the mapping can be viewed in [Additional File 6]. KEGG pathway analysis was performed by mapping the KEGG annotated genes to KEGG pathways as described in the KEGG Mapper tool [68]. Consequently, germ cell specific genes could be identified due to lack of expression in the GCF group, while gonadal somatic genes could be identified due to expression in both the GCF and WT group.

### Phylogeny and synteny

Phylogeny and chromosomal synteny were investigated for *inha* to confirm the identity of this gene. For the phylogenetic analysis, Inha protein sequences from the following species were retrieved from https://www.ncbi.nlm.nih.gov/: Atlantic salmon (GenBank XP_014007683.1), rainbow trout (GenBank XP_021466674.1 (1) and NP_001117672.1 (2)), zebrafish (*Danio rerio*) (GenBank NP_001038669.1), Japanese medaka (*Oryzias latipes*) (GenBank XP_020564073.1), Common carp (*Cyprinus carpio*) (GenBank XP_018966331.1), Ballan wrasse (*Labrus bergylta*) (GenBank XP_020497817.1), Atlantic herring (*Clupea harengus*) (GenBank XP_012676518.1), Spotted gar (*Lepisosteus oculatus*) (GenBank XP_015214647.1), Chicken (*Gallus gallus*) (GenBank NP_001026428.1) and *Xenopus* (GenBank NP_001027522.1 (1), OCT63342.1 (2) and AAI70257.1 (3)). The sequences were imported into MEGA version X [69], and sequence alignments were performed using MUSCLE (Multiple Sequence Comparison by Log-expectation). All positions containing gaps and missing data were eliminated. The phylogenetic analysis was performed applying the Neighbor-Joining method [70]. A bootstrap test [71] was used to test the reliability of the inferred trees.

For the chromosomal synteny, BLAST searches for orthologous genes for salmon *inha* (GenBank 106,575,603) were performed at https://www.ncbi.nlm.nih.gov/. The following fish species were included in the analysis: rainbow trout (Genbank 110,528,842 and 100,135,804), zebrafish (GenBank 570,520), Japanese medaka (GenBank 101,157,430) and Ballan wrasse (GenBank 109,990,152). The Atlantic salmon sequences and annotation were obtained from the official genome annotation (NCBI *Salmo salar* Annotation Release 100).

### RNA extraction, cDNA synthesis and real-time, quantitative PCR

For the group 1 samples, RNA extraction was performed using the MiRNeasy Mini kit (Qiagen), according to the manufacturer’s instructions. Up to 50 mg tissue was used for each sample, and the extracted RNA had absorbance ratios 260/280 of 1,9–2,1 (NanoDrop Spectrophotometer/ThermoFisher Scientifics), and RNA intergrity numbers 7,6–9 (Bioanalyzer/Agilent Technologies). For the group 2 and 3 samples, RNA was extracted and DNase-treated as described previously ([9, 22], respectively). cDNA was synthesized from 125 ng RNA (group 2 samples) and 500 ng RNA (group 3 samples) using the Superscript VILO cDNA synthesis kit (Invitrogen), according to the manufacturer’s instructions. Primers and probe sequences for *inha* and *gsdf* were designed online (https://www.genscript.com/ssl-bin/app/primer (Genscript®)), and can be seen in Table 3. Primers and probe sequences for the housekeeping gene *elongation factor 1-alpha* (*ef1α*) were published previously [72]. QPCR was performed in duplicates in 384-well optical plates in a QuantStudio 5 Real-Time PCR system (ThermoFisher Scientific) (all group 3 samples, and group 2 samples for *gsdf*) or SDS 7900HT Fast Real-Time PCR system (Applied Biosystems) (group 2 samples for *inha*) using default settings. One μl cDNA was used in a 5 (*gsdf*) or 10 μl (*inha*) Fast Taqman qPCR reaction (ThermoFisher Scientific). No-template controls for each gene were run in all qPCR plates. The relative gene expression level was calculated using the comparative Ct (or 2^−ΔΔCt^) method. All values were normalized to *ef1a* and calibrated to the average ΔCt of the testis tissue (group 2 samples) or the immature WT gonads (group 3 samples).

**Table 3:** Primer and probe sequences

Gene	Forward primer	Reverse primer	Probe	Application
*gsdf*	GGCAGCATTTCAGACCACTA	GACAAAGCAGTGGCTGTACC	TGCTGCAGGACCCTCAGCCTGG	qPCR
	atttaggtgacactatagGGTGAGGGTGCTGAACTCAT	taatacgactcactatagggTGCCATGGAGAGTTGTTGAA		ISH probe synthesis
*inha*	TGGTGGCTCTCTCCTCTGAT	ATGAGCAAGTCATCCTCTTCC	CCAGCTCTGGCTCTACCTGTGATAGCT	qPCR
	atttaggtgacactatagGTAGGTGGTCCAGCCATCAG	taatacgactcactatagggTTGGACTGGTTCAAACAGCA		ISH probe synthesis

Primer and probe sequences (5′-3′) designed for *gsdf* and *inha* and used in this study. Sp6 and T7 sequences are shown in lower case letters.

### In situ hybridization

A PCR product was generated using *gsdf* or *inha* gene-specific primers (Table 3) for the synthesis of cRNA-probes for ISH. The PCR products were sequenced following a PCR cleanup using illustra ExoProStar 1-Step (GE Healthcare Life Sciences), according to the manufacturer’s instructions. The returned sequences were blasted in the NCBI database (https://www.ncbi.nlm.nih.gov/) against several species, confirming that the primers had amplified the *gsdf* and *inha* genes. ISH cRNA antisense and sense probes were synthesized from 1 μg PCR product applying primers containing Sp6 or T7 sequence, respectively (Table 3), together with a digoxigenin-alkaline phosphatase (DIG-AP) RNA Labeling Kit (SP6/T7) (Roche Diagnostics). The probes were precipitated, washed, and resuspended in MilliQ water as described by Weltzien et al. [73]. Probe size and quality were checked with a Bioanalyzer (Agilent Technologies), and the DIG incorporation in the probes was inspected by performing a spot-test. ISH by DIG-AP was performed as described by Weltzien et al. [73].

### Statistics

Statistical tests were performed using GraphPad Prism 7.02 (GraphPad Software Inc.). All qPCR datasets were tested for normal distribution using a D’Agostino & Pearson omnibus normality test. For datasets with no normal distribution, or too few n to test for normal distribution, non-parametric tests were performed to calculate differences between groups. A Kruskal-Wallis with Dunn’s multiple comparisons post test was applied for *inha* in different stages of ovary tissue (Fig. 5c), and expression of *gsdf* and *inha* in multi tissues of adult immature salmon (Fig. 4). An ordinary one-way ANOVA with Tukey’s multiple comparisons post test was applied for *gsdf* and *inha* expression in different stages of testis tissue (Fig. 5b, d), and *gsdf* in ovary (Fig. 5a).

## Supplementary information

**Additional file 1.** Gonad specific genes. List of gonad specific (≤50 reads in extragonadal tissues and ≥ 100 reads in ovary and testis) genes in adult Atlantic salmon. Transcript ID, Gene ID, number of reads in multiple tissues (brain, eye, gill, gut, head kidney, heart, kidney, liver, muscle, nose, pyloric caecum, skin, spleen, ovary, testis (GenBank GBRB00000000.1 [23];) are shown

**Additional file 2. **Gonad specific genes with expression preferentially within germ cells. List of gonad specific (≤50 reads in extragonadal tissues and ≥ 100 reads in ovary and testis) genes with expression preferentially within germ cells (≤50 reads in germ cell-free testis and ≥ 100 reads in WT testis) of adult Atlantic salmon males. Transcript ID, Gene ID, number of reads in multiple tissues (brain, eye, gill, gut, head kidney, heart, kidney, liver, muscle, nose, pyloric caecum, skin, spleen, ovary, testis (GenBank GBRB00000000.1 [23]; (highlighted in grey)), and number of reads in juvenile wild type (WT) and *dead end* knockout (*dnd*-KO) testis tissue (highlighted in green) are shown

**Additional file 3.** KEGG pathways. Full list of KEGG pathways annotated to gonad specific genes with expression preferentially within germ cells [Additional File 2] of adult Atlantic salmon males. Pathway ID, pathway, number of genes within each pathway, gene ID and symbol are shown

**Additional file 4. **Phylogenetic analysis of Inha protein sequences. Phylogenetic analysis of Inha protein sequences from Atlantic salmon (GenBank XP_014007683.1), rainbow trout (GenBank XP_021466674.1 (1) and NP_001117672.1 (2)), zebrafish (*Danio rerio*) (GenBank NP_001038669.1), Japanese medaka (*Oryzias latipes*) (GenBank XP_020564073.1), Common carp (*Cyprinus carpio*) (GenBank XP_018966331.1), Ballan wrasse (*Labrus bergylta*) (GenBank XP_020497817.1), Atlantic herring (*Clupea harengus*) (GenBank XP_012676518.1), Spotted gar (*Lepisosteus oculatus*) (GenBank XP_015214647.1), Chicken (*Gallus gallus*) (GenBank NP_001026428.1) and Xenopus (GenBank NP_001027522.1 (1), OCT63342.1 (2) and AAI70257.1 (3)). The evolutionary history was inferred using the Neighbor-Joining method [70]. The bootstrap consensus tree inferred from 500 replicates is taken to represent the evolutionary history of the taxa analyzed [71]. Branches corresponding to partitions reproduced in less than 50% bootstrap replicates are collapsed. The percentage of replicate trees in which the associated taxa clustered together in the bootstrap test are shown next to the branches [71]. The evolutionary distances were computed using the Poisson correction method [74] and are in the units of the number of amino acid substitutions per site. This analysis involved 13 amino acid sequences. All ambiguous positions were removed for each sequence pair (pairwise deletion option). A total of 432 positions were in the final dataset. Evolutionary analyses were conducted in MEGA X [69]

**Additional file 5.** ARRIVE Guidelines Checklist. Completed “The ARRIVE Guidelines Checklist” for reporting information on experimental animals in this study

**Additional file 6.** RNA seq mapping summary. Summary of the mapping of RNA-seq paired end sequences against the gene model transcripts of the Atlantic salmon genome (ICSASG_v2). Sample name, total reads, mapped reads and % mapping is shown

## Data Availability

The raw RNA sequencing data are available at https://www.ncbi.nlm.nih.gov/bioproject/PRJNA550414. The gene lists resulting from filtering of RNA sequencing data are available in the published paper and its additional files.

## Abbreviations

GCF: Germ cell-free
ISH: In situ hybridization
TGF-β: Transforming Growth Factor
WT: Wild type

## References

[1] Glover KA, Pertoldi C, Besnier F, Wennevik V, Kent M, Skaala O (2013). Atlantic salmon populations invaded by farmed escapees: quantifying genetic introgression with a Bayesian approach and SNPs. BMC Genet.

[2] Glover KA, Quintela M, Wennevik V, Besnier F, Sørvik AG, Skaal O (2012). Three decades of farmed escapees in the wild: a spatio-temporal analysis of Atlantic salmon population genetic structure throughout Norway. PLoS One.

[3] Wargelius A, Leininger S, Skaftnesmo KO, Kleppe L, Andersson E, Taranger GL, Schulz RW, Edvardsen RB (2016). Dnd knockout ablates germ cells and demonstrates germ cell independent sex differentiation in Atlantic salmon. Sci Rep.

[4] Yoshizaki G, Takashiba K, Shimamori S, Fujinuma K, Shikina S, Okutsu T, Kume S, Hayashi M (2016). Production of germ cell-deficient salmonids by dead end gene knockdown, and their use as recipients for germ cell transplantation. Mol Reprod Dev.

[5] Wong TT, Zohar Y (2015). Production of reproductively sterile fish by a non-transgenic gene silencing technology. Sci Rep.

[6] Wong TT, Zohar Y (2015). Production of reproductively sterile fish: a mini-review of germ cell elimination technologies. Gen Comp Endocrinol.

[7] Nagasawa K, Fernandes JM, Yoshizaki G, Miwa M, Babiak I (2013). Identification and migration of primordial germ cells in Atlantic salmon, *Salmo salar*: characterization of *vasa*, *dead end*, and *lymphocyte antigen 75* genes. Mol Reprod Dev.

[8] Kleppe L, Wargelius A, Johnsen H, Andersson E, Edvardsen RB (2015). Gonad specific genes in Atlantic salmon (*Salmo salar L*.): characterization of *tdrd7-2*, *dazl-2*, *piwil1* and *tdrd1* genes. Gene.

[9] Kleppe L, Edvardsen RB, Furmanek T, Andersson E, Juanchich A, Wargelius A (2017). *bmp15l*, *figla*, *smc1bl*, and *larp6l* are preferentially expressed in germ cells in Atlantic salmon (*Salmo salar L*.). Mol Reprod Dev.

[10] Yan C, Wang P, DeMayo J, DeMayo FJ, Elvin JA, Carino C, Prasad SV, Skinner SS, Dunbar BS, Dube JL, Celeste AJ, Matzuk MM (2001). Synergistic roles of bone morphogenetic protein 15 and growth differentiation factor 9 in ovarian function. Mol Endocrinol.

[11] McNatty KP, Hudson NL, Whiting L, Reader KL, Lun S, Western A, Heath DA, Smith P, Moore LG, Juengel JL (2007). The effects of immunizing sheep with different BMP15 or GDF9 peptide sequences on ovarian follicular activity and ovulation rate. Biol Reprod.

[12] McIntosh CJ, Lawrence S, Smith P, Juengel JL, McNatty KP (2012). Active immunization against the proregions of GDF9 or BMP15 alters ovulation rate and litter size in mice. Reproduction.

[13] Juengel JL, Proctor LE, Wearne K, Olliver D, Hudson NL, Jensen D, Davis GH, Johnstone PD, McNatty KP (2013). Effects of immunization against androstenedione or bone morphogenetic protein 15 (BMP15) on reproductive performance in sheep. J Anim Sci.

[14] Lin Q, Mei J, Li Z, Zhang X, Zhou L, Gui JF (2017). Distinct and cooperative roles of *amh* and *dmrt1* in self-renewal and differentiation of Male germ cells in Zebrafish. Genetics.

[15] Yano Ayaka, Guyomard René, Nicol Barbara, Jouanno Elodie, Quillet Edwige, Klopp Christophe, Cabau Cédric, Bouchez Olivier, Fostier Alexis, Guiguen Yann (2012). An Immune-Related Gene Evolved into the Master Sex-Determining Gene in Rainbow Trout, Oncorhynchus mykiss. Current Biology.

[16] Yano A, Nicol B, Jouanno E, Quillet E, Fostier A, Guyomard R, Guiguen Y (2013). The sexually dimorphic on the Y-chromosome gene (*sdY*) is a conserved male-specific Y-chromosome sequence in many salmonids. Evol Appl.

[17] Andersson E, Nijenhuis W, Male R, Swanson P, Bogerd J, Taranger GL, Schulz RW (2009). Pharmacological characterization, localization and quantification of expression of gonadotropin receptors in Atlantic salmon (*Salmo salar L*.) ovaries. Gen Comp Endocrinol.

[18] Maugars G, Schmitz M (2006). Molecular cloning and characterization of FSH and LH receptors in Atlantic salmon (*Salmo salar L*.). Gen Comp Endocrinol.

[19] Chen SX, Bogerd J, Andersson E, Almeida FF, Taranger GL, Schulz RW (2011). Cloning, pharmacological characterization, and expression analysis of Atlantic salmon (*Salmo salar L*.) nuclear progesterone receptor. Reproduction.

[20] von Schalburg KR, Gowen BE, Rondeau EB, Johnson NW, Minkley DR, Leong JS, Davidson WS, Koop BF (2013). Sex-specific expression, synthesis and localization of aromatase regulators in one-year-old Atlantic salmon ovaries and testes. Comp Biochem Physiol B Biochem Mol Biol.

[21] von Schalburg KR, Yasuike M, Yazawa R, de Boer JG, Reid L, So S, Robb A, Rondeau EB, Phillips RB, Davidson WS, Koop BF (2011). Regulation and expression of sexual differentiation factors in embryonic and extragonadal tissues of Atlantic salmon. BMC Genomics.

[22] Kleppe L, Andersson E, Skaftnesmo KO, Edvardsen RB, Fjelldal PG, Norberg B, Bogerd J, Schulz RW, Wargelius A (2017). Sex steroid production associated with puberty is absent in germ cell-free salmon. Sci Rep.

[23] Lien S, Koop BF, Sandve SR, Miller JR, Kent MP, Nome T, Hvidsten TR, Leong JS, Minkley DR, Zimin A (2016). The Atlantic salmon genome provides insights into rediploidization. Nature.

[24] Sawatari E, Shikina S, Takeuchi T, Yoshizaki G (2007). A novel transforming growth factor-beta superfamily member expressed in gonadal somatic cells enhances primordial germ cell and spermatogonial proliferation in rainbow trout (*Oncorhynchus mykiss*). Dev Biol.

[25] Lubieniecki KP, Botwright NA, Taylor RS, Evans BS, Cook MT, Davidson WS (2015). Expression analysis of sex-determining pathway genes during development in male and female Atlantic salmon (*Salmo salar*). Physiol Genomics.

[26] Braasch I, Gehrke AR, Smith JJ, Kawasaki K, Manousaki T, Pasquier J, Amores A, Desvignes T, Batzel P, Catchen J (2016). The spotted gar genome illuminates vertebrate evolution and facilitates human-teleost comparisons. Nat Genet.

[27] Schulz RW, Taranger GL, Bogerd J, Nijenhuis W, Norberg B, Male R, Andersson E (2019). Entry into puberty is reflected in changes in hormone production but not in testicular receptor expression in Atlantic salmon (*Salmo salar*). Reprod Biol Endocrinol.

[28] Young JC, Wakitani S, Loveland KL (2015). TGF-beta superfamily signaling in testis formation and early male germline development. Semin Cell Dev Biol.

[29] Wang H, Yuan Q, Sun M, Niu M, Wen L, Fu H, Zhou F, Chen Z, Yao C, Hou J (2017). BMP6 Regulates Proliferation and Apoptosis of Human Sertoli Cells Via Smad2/3 and Cyclin D1 Pathway and DACH1 and TFAP2A Activation. Sci Rep.

[30] Smith A, Avaron F, Guay D, Padhi BK, Akimenko MA (2006). Inhibition of BMP signaling during zebrafish fin regeneration disrupts fin growth and scleroblasts differentiation and function. Dev Biol.

[31] Neves JV, Caldas C, Wilson JM, Rodrigues PN (2011). Molecular mechanisms of hepcidin regulation in sea bass (*Dicentrarchus labrax*). Fish Shellfish Immunol.

[32] Cleves PA, Ellis NA, Jimenez MT, Nunez SM, Schluter D, Kingsley DM, Miller CT (2014). Evolved tooth gain in sticklebacks is associated with a cis-regulatory allele of Bmp6. Proc Natl Acad Sci U S A.

[33] Cleves PA, Hart JC, Agoglia RM, Jimenez MT, Erickson PA, Gai L, Miller CT (2018). An intronic enhancer of Bmp6 underlies evolved tooth gain in sticklebacks. PLoS Genet.

[34] Ma Q, Feng W, Zhuang Z, Liu S (2017). Cloning, expression profiling and promoter functional analysis of bone morphogenetic protein 6 and 7 in tongue sole (*Cynoglossus semilaevis*). Fish Physiol Biochem.

[35] Li CW, Ge W (2011). Spatiotemporal expression of bone morphogenetic protein family ligands and receptors in the zebrafish ovary: a potential paracrine-signaling mechanism for oocyte-follicle cell communication. Biol Reprod.

[36] Harpelunde Poulsen K, Jorgensen A (2019). Role of nodal signalling in testis development and initiation of testicular cancer. Reproduction.

[37] Tian RH, Yang S, Zhu ZJ, Wang JL, Liu Y, Yao C, Ma M, Guo Y, Yuan Q, Hai Y (2015). NODAL secreted by male germ cells regulates the proliferation and function of human Sertoli cells from obstructive azoospermia and nonobstructive azoospermia patients. Asian J Androl.

[38] Bennett JT, Stickney HL, Choi WY, Ciruna B, Talbot WS, Schier AF (2007). Maternal nodal and zebrafish embryogenesis. Nature.

[39] Turk V, Stoka V, Vasiljeva O, Renko M, Sun T, Turk B, Turk D (2012). Cysteine cathepsins: from structure, function and regulation to new frontiers. Biochim Biophys Acta.

[40] Turk V, Turk B, Turk D (2001). Lysosomal cysteine proteases: facts and opportunities. EMBO J.

[41] Wright WW, Zabludoff SD, Penttilä TL, Parvinen M (1995). Germ cell-Sertoli cell interactions: regulation by germ cells of the stage-specific expression of CP-2/cathepsin L mRNA by Sertoli cells. Dev Genet.

[42] Shibata Y, Paul-Prasanth B, Suzuki A, Usami T, Nakamoto M, Matsuda M, Nagahama Y (2010). Expression of gonadal soma derived factor (GSDF) is spatially and temporally correlated with early testicular differentiation in medaka. Gene Expr Patterns.

[43] Gautier A, Le Gac F, Lareyre JJ (2011). The *gsdf* gene locus harbors evolutionary conserved and clustered genes preferentially expressed in fish previtellogenic oocytes. Gene.

[44] Gautier A, Sohm F, Joly JS, Le Gac F, Lareyre JJ (2011). The proximal promoter region of the zebrafish *gsdf* gene is sufficient to mimic the spatio-temporal expression pattern of the endogenous gene in Sertoli and granulosa cells. Biol Reprod.

[45] Kaneko H, Ijiri S, Kobayashi T, Izumi H, Kuramochi Y, Wang DS, Mizuno S, Nagahama Y (2015). *Gonadal soma-derived factor* (*gsdf*), a TGF-beta superfamily gene, induces testis differentiation in the teleost fish *Oreochromis niloticus*. Mol Cell Endocrinol.

[46] Liu Y, Zhang W, Du X, Zhao J, Liu X, Li X, Zhang Q, Wang X (2017). Sexually dimorphic expression in developing and adult gonads shows an important role of *gonadal soma-derived factor* during sex differentiation in olive flounder (*Paralichthys olivaceus*). Comp Biochem Physiol B Biochem Mol Biol.

[47] Jiang DN, Mustapha UF, Shi HJ, Huang YQ, Si-Tu JX, Wang M, Deng SP, Chen HP, Tian CX, Zhu CH (2019). Expression and transcriptional regulation of *gsdf* in spotted scat (*Scatophagus argus*). Comp Biochem Physiol B Biochem Mol Biol.

[48] Yang Y, Liu Q, Xiao Y, Xu S, Wang X, Yang J, Song Z, You F, Li J (2019). High temperature increases the *gsdf* expression in masculinization of genetically female Japanese flounder (*Paralichthys olivaceus*). Gen Comp Endocrinol.

[49] Zhu Y, Meng L, Xu W, Cui Z, Zhang N, Guo H, Wang N, Shao C, Chen S (2018). The autosomal Gsdf gene plays a role in male gonad development in Chinese tongue sole (*Cynoglossus semilaevis*). Sci Rep.

[50] Myosho Taijun, Otake Hiroyuki, Masuyama Haruo, Matsuda Masaru, Kuroki Yoko, Fujiyama Asao, Naruse Kiyoshi, Hamaguchi Satoshi, Sakaizumi Mitsuru (2012). Tracing the Emergence of a Novel Sex-Determining Gene in Medaka, Oryzias luzonensis. Genetics.

[51] Imai T, Saino K, Matsuda M (2015). Mutation of *Gonadal soma-derived factor* induces medaka XY gonads to undergo ovarian development. Biochem Biophys Res Commun.

[52] Zhang X, Guan G, Li M, Zhu F, Liu Q, Naruse K, Herpin A, Nagahama Y, Li J, Hong Y (2016). Autosomal *gsdf* acts as a male sex initiator in the fish medaka. Sci Rep.

[53] Jiang DN, Yang HH, Li MH, Shi HJ, Zhang XB, Wang DS (2016). *gsdf* is a downstream gene of *dmrt1* that functions in the male sex determination pathway of the Nile tilapia. Mol Reprod Dev.

[54] Horiguchi R, Nozu R, Hirai T, Kobayashi Y, Nagahama Y, Nakamura M (2013). Characterization of *gonadal soma-derived factor* expression during sex change in the protogynous wrasse, Halichoeres trimaculatus. Dev Dyn.

[55] Zhu Y, Wang C, Chen X, Guan G (2016). Identification of *gonadal soma-derived factor* involvement in *Monopterus albus* (protogynous rice field eel) sex change. Mol Biol Rep.

[56] Guan G, Sun K, Zhang X, Zhao X, Li M, Yan Y, Wang Y, Chen J, Yi M, Hong Y (2017). Developmental tracing of oocyte development in *gonadal soma-derived factor* deficiency medaka (*Oryzias latipes*) using a transgenic approach. Mech Dev.

[57] Yan YL, Desvignes T, Bremiller R, Wilson C, Dillon D, High S, Draper B, Buck CL, Postlethwait J (2017). Gonadal soma controls ovarian follicle proliferation through Gsdf in zebrafish. Dev Dyn.

[58] Wu X, Zhang Y, Xu S, Chang Y, Ye Y, Guo A, Kang Y, Guo H, Xu H, Chen L (2019). Loss of Gsdf leads to a dysregulation of Igf2bp3-mediated oocyte development in medaka. Gen Comp Endocrinol.

[59] Luckenbach JA, Iliev DB, Goetz FW, Swanson P. Identification of differentially expressed ovarian genes during primary and early secondary oocyte growth in coho salmon, *Onchorhynchus kisutch*. Reprod Biol Endocrinol. 2008;6(2):1477–7827. 10.1186/1477-7827-6-2 PMC2262088 18205936

[60] Tada T, Endo M, Hirono I, Takashima F, Aoki T (2002). Differential expression and cellular localization of *activin* and *inhibin* mRNA in the rainbow trout ovary and testis. Gen Comp Endocrinol.

[61] Poon SK, So WK, Yu X, Liu L, Ge W (2009). Characterization of *inhibin alpha subunit* (*inha*) in the zebrafish: evidence for a potential feedback loop between the pituitary and ovary. Reproduction.

[62] Guzman JM, Luckenbach JA, Yamamoto Y, Swanson P (2014). Expression profiles of Fsh-regulated ovarian genes during oogenesis in coho salmon. PLoS One.

[63] Sambroni E, Rolland AD, Lareyre JJ, Le Gac F (2012). FSH and LH have common and distinct effects on gene expression in rainbow trout testis. J Mol Endocrinol.

[64] Sambroni E, Lareyre JJ, Le Gac F (2013). Fsh controls gene expression in fish both independently of and through steroid mediation. PLoS One.

[65] Fjelldal PG, Hansen T, Huang T (2011). Continuous light and elevated temperature can trigger maturation both during and immediately after smoltification in male Atlantic salmon (*Salmo salar*). Aquaculture.

[66] Langmead B, Salzberg SL (2012). Fast gapped-read alignment with bowtie 2. Nat Methods.

[67] Li H, Handsaker B, Wysoker A, Fennell T, Ruan J, Homer N, Marth G, Abecasis G, Durbin R (2009). 1000 genome project data processing subgroup. The sequence alignment/map format and SAMtools. Bioinformatics.

[68] Aoki-Kinoshita KF, Kanehisa M (2007). Gene annotation and pathway mapping in KEGG. Methods Mol Biol.

[69] Kumar S, Stecher G, Li M, Knyaz C, Tamura K (2018). MEGA X: molecular evolutionary genetics analysis across computing platforms. Mol Biol Evol.

[70] Saitou N, Nei M (1987). The neighbor-joining method: a new method for reconstructing phylogenetic trees. Mol Biol Evol.

[71] Felsenstein J (1985). Confidence limits on phylogenies: an approach using the bootstrap. Evolution.

[72] Olsvik PA, Lie KK, Jordal AE, Hordvik I, Nilsen TO (2005). Evaluation of potential reference genes in real-time RT-PCR studies of Atlantic salmon. BMC Mol Biol.

[73] Weltzien FA, Norberg B, Helvik JV, Andersen O, Swanson P, Andersson E (2003). Identification and localization of eight distinct hormone-producing cell types in the pituitary of male Atlantic halibut (*Hippoglossus hippoglossus L*.). Comp Biochem Physiol A Mol Integr Physiol.

[74] Zuckerkandl L, Pauling L (1965). Evolutionary divergence and convergence in proteins.

