# Supplementary figures and images for: Transcriptomic analysis of dead end knockout testis reveals germ cell and gonadal somatic factors in Atlantic salmon
Source: BMC Genomics. 2020 Jan 30;21:99. doi: 10.1186/s12864-020-6513-4 (PMC6993523; doi:10.1186/s12864-020-6513-4)

#### Additional File 4 - Phylogenetic analysis of Inha protein sequences

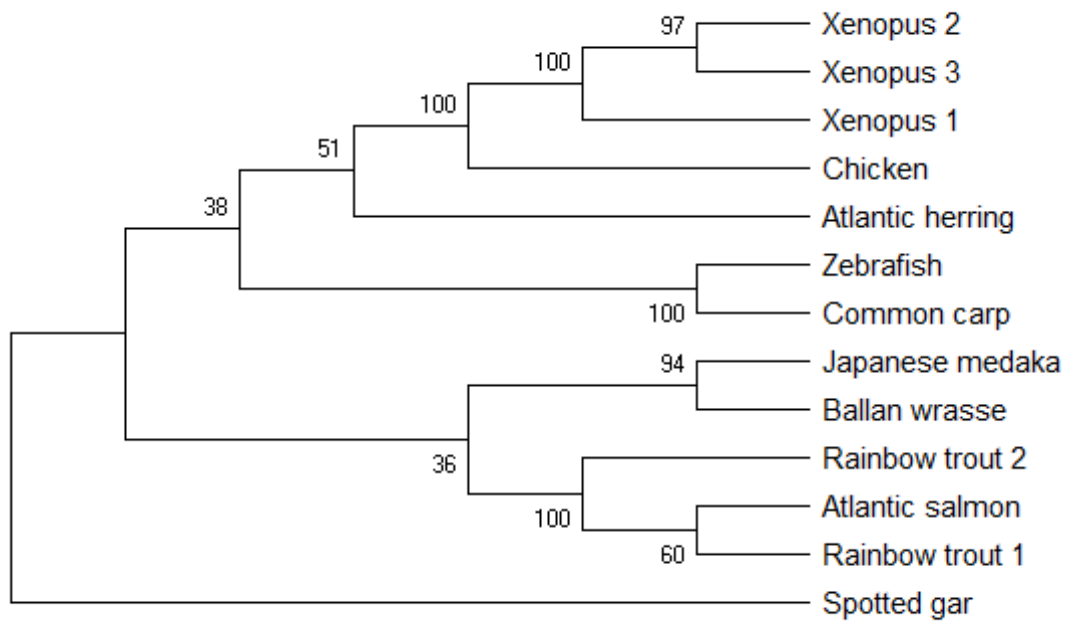

Supplement: Supplementary file 4 — Additional file 4. Phylogenetic analysis of Inha protein sequences. Phylogenetic analysis of Inha protein sequences from Atlantic salmon (GenBank XP_014007683.1), rainbow trout (GenBank XP_021466674.1 (1) and NP_001117672.1 (2)), zebrafish (Danio rerio) (GenBank NP_001038669.1), Japanese medaka (Oryzias latipes) (GenBank XP_020564073.1), Common carp (Cyprinus carpio) (GenBank XP_018966331.1), Ballan wrasse (Labrus bergylta) (GenBank XP_020497817.1), Atlantic herring (Clupea harengus) (GenBank XP_012676518.1), Spotted gar (Lepisosteus oculatus) (GenBank XP_015214647.1), Chicken (Gallus gallus) (GenBank NP_001026428.1) and Xenopus (GenBank NP_001027522.1 (1), OCT63342.1 (2) and AAI70257.1 (3)). The evolutionary history was inferred using the Neighbor-Joining method [70]. The bootstrap consensus tree inferred from 500 replicates is taken to represent the evolutionary history of the taxa analyzed [71]. Branches corresponding to partitions reproduced in less than 50% bootstrap replicates are collapsed. The percentage of replicate trees in which the associated taxa clustered together in the bootstrap test are shown next to the branches [71]. The evolutionary distances were computed using the Poisson correction method [74] and are in the units of the number of amino acid substitutions per site. This analysis involved 13 amino acid sequences. All ambiguous positions were removed for each sequence pair (pairwise deletion option). A total of 432 positions were in the final dataset. Evolutionary analyses were conducted in MEGA X [69] [file 12864_2020_6513_MOESM4_ESM.pdf]
