# Supplementary material for: Transcriptomic analysis of dead end knockout testis reveals germ cell and gonadal somatic factors in Atlantic salmon
Source: BMC Genomics. 2020 Jan 30;21:99. doi: 10.1186/s12864-020-6513-4 (PMC6993523; doi:10.1186/s12864-020-6513-4)
Supplement: Supplementary file 6 — Additional file 6. RNA seq mapping summary. Summary of the mapping of RNA-seq paired end sequences against the gene model transcripts of the Atlantic salmon genome (ICSASG_v2). Sample name, total reads, mapped reads and % mapping is shown [file 12864_2020_6513_MOESM6_ESM.pdf]

Additional File 6 – RNA seq mapping summary

Sample	Total reads	Mapped reads	Mapping
control_6-Tes_5	72402608	52357592	72,31 %
control_7-Tes_6	38146482	26435474	69,30 %
control_9-Tes_3E	90410402	62482125	69,11 %
dndKO_3-Tes_8G	47417012	33366148	70,37 %
dndKO_4-Tes_1	75854722	59427438	78,34 %
dndKO_5-Tes_2	76327982	57690447	75,58 %
dndKO_8-Tes_2E	55143820	40315717	73,11 %
